# Assessing Professionals’ Adoption Readiness for eMental Health: Development and Validation of the eMental Health Adoption Readiness Scale

**DOI:** 10.2196/28518

**Published:** 2021-09-17

**Authors:** Milou A Feijt, Yvonne A W de Kort, Joyce H D M Westerink, Joyce J P A Bierbooms, Inge M B Bongers, Wijnand A IJsselsteijn

**Affiliations:** 1 Human-Technology Interaction Group Department of Industrial Engineering & Innovation Sciences Eindhoven University of Technology Eindhoven Netherlands; 2 Digital Engagement, Cognition & Behavior Group Philips Research Eindhoven Netherlands; 3 Tranzo Tilburg School of Social and Behavioural Sciences Tilburg University Tilburg Netherlands; 4 Mental Healthcare Eindhoven Eindhoven Netherlands

**Keywords:** eMental health, adoption of innovation, mental health care, scale development

## Abstract

**Background:**

The last few decades have witnessed significant advances in the development of digital tools and applications for mental health care. Despite growing evidence for their effectiveness, acceptance and use of these tools in clinical practice remain low. Hence, a validated and easy-to-use instrument for assessing professionals’ readiness to adopt eMental health (EMH) is necessary to gain further insights into the process of EMH adoption and facilitate future research on this topic.

**Objective:**

The aim of this study is to develop and validate an instrument for assessing mental health care professionals’ readiness to adopt EMH.

**Methods:**

Item generation was guided by literature and inputs from mental health care professionals and experts in survey development. Exploratory factor analyses were conducted on an initial set of 29 items completed by a sample of mental health care professionals (N=432); thereafter, the scale was reduced to 15 items in an iterative process. The factor structure thus obtained was subsequently tested using a confirmatory factor analysis with a second sample of mental health care professionals (N=363). The internal consistency, convergent validity, and predictive validity of the eMental Health Adoption Readiness (eMHAR) Scale were assessed.

**Results:**

Exploratory factor analysis resulted in a 3-factor solution with 15 items. The factors were analyzed and labeled as perceived benefits and applicability of EMH, EMH proactive innovation, and EMH self-efficacy. These factors were confirmed through a confirmatory factor analysis. The total scale and subscales showed a good internal consistency (Cronbach *α*=.73-.88) along with acceptable convergent and predictive relationships with related constructs.

**Conclusions:**

The constructed eMHAR Scale showed a conceptually interpretable 3-factor structure having satisfactory characteristics and relationships with relevant concepts. Its ease of use allows for quick acquisition of data that can contribute to understanding and facilitating the process of adoption of EMH by clinical professionals.

## Introduction

Over recent decades, a wide range of digital tools and technologies have been used in the practice of mental health care. There is a growing body of evidence for the effectiveness of eMental health (EMH) [1,2], which can be defined as “the use of information and communication technolog*y* (ICT)*—*in particular, the many technologies related to the Internet—when these technologies are used to support and improve mental health conditions and mental healthcare” [3]*.* Multiple benefits, such as an increased access to psychological treatment, convenience, and enhanced self-reflection and self-disclosure of the client, are associated with the use of these technologies [4-7]. Despite these advantages, studies consistently report that, before COVID-19, the use of EMH in daily practice was relatively low, finding that 80%-90% of practitioners never or only rarely made use of web-based tools in their routine [8-10].

Several studies have aimed to elucidate what determines this low use of EMH and have pointed to a variety of factors that can play a role, such as the characteristics of clients and professionals, technological aspects, and legal and managerial issues [11-13]. Initially, it was assumed that client factors such as their attitudes and skills were the most important barriers; therefore, studies tended to focus on clarifying these characteristics [14]. However, research suggests that the role of professionals is at least as important and that their adoption is crucial in the successful implementation of EMH [15]. Adoption has been described as a staged process that comprises required knowledge and skills, acceptance, implementation in daily procedures, actual use, and evaluation [16]. Reviews on the perspective of professionals demonstrate that a variety of factors influence adoption, such as (lack of) perceived benefits, applicability and conditions in daily practice, experience and know-how regarding EMH, and technical issues [6,7,17].

As conceptualized in the Levels of Adoption of eMental Health Model [5], each stage in the adoption process is associated with different barriers and drivers that professionals experience. Some of these are external factors, such as the organizational setting and supporting conditions in daily practice (eg, time and resources provided by an institution), characteristics of the EMH tool, and available technical infrastructure, whereas other factors concern individual characteristics of professionals, such as their beliefs and attitudes, knowledge, skills, and experience regarding EMH [12]. Research has shown that these individual characteristics are crucial to the adoption process [13]. Therefore, in this study, we focus on these personal characteristics and refer to these with the term adoption readiness—the extent to which a professional is ready to use EMH (ie, has a positive attitude, is motivated, and possesses the necessary skills and knowledge). By doing so, we distinguish individual adoption readiness from the broader context of adoption, as the latter also includes the actual use in practice and hence is highly influenced by external factors [12].

Studies that investigated relevant individual characteristics generally reported relatively low levels of acceptance, skills, knowledge, and experience regarding EMH, that is, low adoption readiness [6,11,13]. These studies also indicated that perceived benefits and applicability in daily practice, an innovative attitude, experience, and feelings of self-competency regarding EMH are important determinants of adoption readiness. Although providing valuable knowledge on adoption readiness, each of these studies used different measures and definitions; to date, no validated quantitative instrument comprehensively captures all the relevant aspects. This complicates research on this topic, and professionals’ adoption readiness and its underlying factors have remained difficult to qualify and quantify, which also makes it more difficult to develop effective strategies to increase the uptake of EMH.

To improve our understanding, we need a measurement instrument that can reliably assess a professional’s readiness to adopt EMH. The development of our scale benefits from previous studies in the same direction. The measurement instruments that were used in these studies focused on attitudes [18], comprised *ad hoc* measures for one specific study [19,20], or were measures that were originally developed to assess individuals’ adoption of technology in general [6,9,10]. Other studies have used qualitative methods that have the strength of providing in-depth results, but are less suited for studying larger sample sizes and cross-group comparisons [21,22]. In addition, related studies have focused on the client’s perception [23] or organizational structures and implementation strategies [24]. We build upon and extend existing measures by developing a validated scale that specifically focuses on gauging individuals’ readiness to adopt new or existing EMH tools, independent of organizational settings or specific tools. A practical measurement instrument would facilitate studies with larger samples and allow scholars to systematically test the specific hypotheses about how professionals’ adoption readiness relates to external factors, how the various individual and external factors interact, and what their relative roles are in the actual use of EMH in mental health care practice. In addition, it would allow cross-study comparisons to investigate the differences between demographic groups and longitudinal comparisons to examine developments over time—all important means to increase our understanding of the EMH adoption process.

This study presents the process of the development and validation of the eMental Health Adoption Readiness (eMHAR) Scale: a scale that assesses EMH adoption readiness of mental health care professionals. Specifically, this study describes the scale construction process (item generation and item selection), and validation of the scale through an exploratory factor analysis followed by a confirmatory factor analysis, using two sizable samples of mental health care professionals, along with an analysis of scale reliability as well as convergent and predictive validity. In the *Discussion* section, we will highlight the relevance of the scale and reflect on its theoretical and applied strengths and current limitations.

## Methods

### Scale Construction

The construction of the eMHAR Scale broadly involved the following steps: (1) item development, generating an item pool, and having survey experts review the items; (2) test with a sample of the target population to explore the underlying factor structure, refine the scale, and obtain an indication of the psychometric properties of the resulting scale [25,26]; and (3) a second study with a different sample to confirm the factor structure and further establish its psychometric properties. This procedure of first conducting an exploratory factor analysis followed by a confirmatory factor analysis is generally recommended in scale development [27-29]. The next sections describe each of these steps in more detail. The study protocol was assessed and approved by the ethical review board of the Eindhoven University of Technology.

### Item Development

To generate our item pool, we started with the results of Feijt et al [5]. This qualitative study reported factors associated with EMH adoption readiness that formed the basis for the items that should be covered by the scale. These factors included perceived benefits, applicability in daily clinical practice, personal affinity, proactive behavior, knowledge, experience, and skills. To ensure that we did not miss any relevant topics, we additionally searched PsycINFO and PubMed for articles on EMH adoption readiness that were published since the publication of this work [5]. Thus, we used the terms (*e(Mental)Health* OR *technology*) AND (*adoption* OR *attitude* OR *barriers* OR *drivers*) and limited the results to articles published from April 2017 to January 2018. This search supported our findings and did not yield additional topics.

We translated the topics into statements that used the language familiar to mental health care professionals. This procedure led to the first draft version of the eMHAR Scale. It comprised 25 statements with a 5-point Likert scale response format: strongly disagree, disagree, neutral, agree, and strongly agree, which were assigned the values 1-5, respectively. We collected feedback on this draft version from 20 mental health care professionals as part of a workshop that took place during an EMH event. With 3 of them, we subsequently used a (phone-based) think-aloud strategy while filling out the questionnaire. Thus, we obtained a better understanding of unclear or redundant items. On the basis of the feedback, several items were rephrased, and definitions were clarified: four new items were added (three regarding pioneering activities and one on perceived added value) and two items that were considered duplications (regarding the lack of applicability of EMH and experience with EMH) were removed. This second draft version was presented to 3 experts on the topic of EMH for a final check to ensure that all the identified aspects of EMH adoption readiness were adequately and sufficiently covered. On the basis of this expert review, another two items were added to skills and feelings of competency, further resulting in a final item pool of 29 items (Multimedia Appendix 1).

### Validation Study 1

#### Web-Based Survey

The data for the first validation were collected via a web-based survey. In addition to the eMHAR Scale items, the survey comprised self-developed items on concepts related to adoption readiness of EMH to analyze convergent validity: perceived added value, feeling of competency regarding EMH in general, perceived proficiency for various EMH skills and for specific tools, and frequency of use. *The perceived added value* of EMH was measured for each of 13 EMH tools (eg, videocall, web-based modules, and virtual reality) on 5-point scales ranging from 1 (not valuable) to 5 (very valuable). *Feeling of competency* regarding EMH was measured using a 5-point scale describing incrementally increasing levels of skills and knowledge. Moreover, participants indicated *perceived proficiency* regarding nine EMH skills (eg, ability to establish an empathic interaction on the internet and having sufficient knowledge of privacy and security requirements) and *perceived proficiency* regarding the same 13 EMH tools on 5-point scales ranging from 1 (not at all competent) to 5 (very competent). Last, the *frequency of use of EMH* was also probed for the same 13 EMH tools on 5-point scales from 1 (almost never) to 5 (almost every day). The items for these convergent measures can be found in Multimedia Appendix 2. We expected that the eMHAR Scale or its potential subscales would show substantial positive correlations with these constructs.

Finally, the survey included basic demographic questions and several items regarding the characteristics of participants' everyday clinical practice (ie, which psychological disorders they treated, which kinds of psychological treatments they provided, years of professional experience, and whether they had received training in EMH). The time required to complete the survey was approximately 15 minutes.

#### Sample Size and Recruitment

For factor analysis, a sufficient observation-to-item ratio is required to ensure the reliability of the correlation coefficients that are used to compute the factors [30]. Although there are varying opinions on what exactly is an adequate ratio, an index of 10:1 is generally considered sufficient [29,30]. With our item pool of 29 items, our aim was to recruit at least 300 mental health care professionals. The target sample comprised mental health care professionals representing broader ranges in the use and experience of EMH, in the types of professions within mental health care, and the types of mental health care institutions to which they were affiliated. To achieve this, the participants were recruited by contacting several large mental health care organizations spread across the Netherlands. A total of six mental health care institutions disseminated an announcement with information and the survey link either through email or intranet. Furthermore, a similar announcement was placed in the newsletters and on the network webpages of three national professional associations of psychologists: the Dutch Institute for Psychology, the Dutch Association of Health Psychologists, and the Dutch Association of Independent Psychotherapists. In addition, several independent practitioners were contacted directly through the authors’ network. Participants could sign up for a raffle in which 10 gift vouchers were allotted as a reward for their participation.

#### Data Collection and Sample

Data were collected between October 2018 and April 2019. In total, 432 participants (288/432, 66.7% female) completed the survey, with ages ranging from 20 to 69 years (mean 41.3, SD 12.1). The most frequently reported professions were clinical or counseling psychologists, psychiatric nurses, and social workers. Table 1 presents further details of the demographic data of the sample.

#### Data Screening

Before the factor analysis, the items were examined for the accuracy of data entry, missing values, and their fit with multivariate assumptions by checking their distributions, descriptive statistics, frequencies, plots, and standardized residuals [30]. Second, the existence of multivariate outliers was examined with the Mahalanobis distance at *P*<.001, which was evaluated as chi-square with the degrees of freedom equal to the number of variables [30]. Identified cases were inspected to understand the cause of the significant value, and analyses were both run with and without the outliers to determine the effect of the outliers on the results, to decide upon their retention or removal.

**Table 1:** Demographic data for the samples of study 1 (N=432) and study 2 (N=363), including gender, age, and profession.

Characteristic			Study 1, n (%)		Study 2, n (%)
**Gender**					
	Male	144 (33.3)		95 (26.2)	
	Female	288 (66.7)		268 (73.8)	
**Age (years)**					
	<25	31 (7.2)		24 (6.6)	
	26-35	144 (33.3)		149 (41.0)	
	36-45	83 (19.2)		83 (22.8)	
	46-55	106 (24.5)		65 (17.9)	
	56-65	63 (14.6)		40 (11.0)	
	65+	2 (0.5)		2 (0.6)	
**Profession**					
	Clinical or counseling psychologists and psychotherapists	126 (29.2)		135 (37.2)	
	Psychiatric nurses	147 (34.0)		118 (32.5)	
	Social work	115 (26.6)		78 (21.5)	
	Expressive therapists (eg, creative arts therapist and psychomotor therapist)	13 (3.0)		8 (2.2)	
	Physicians (eg, psychiatrist, general practitioner, and neurologist)	27 (6.3)		23 (6.3)	
	Other (eg, researcher and team manager)	4 (0.9)		1 (0.3)	

#### Scale Analysis and Evaluation

Although we had expectations about the underlying factors of the eMHAR Scale based on a previous work [5], we conducted an exploratory factor analysis to allow for the detection of nonhypothesized factors, which is a recommended procedure in the early stages of scale development [27,28,31]. Owing to the ordinal nature of Likert scales, the factor analysis in this study was conducted on the polychoric correlation matrix to correct for statistical issues that could arise because of the attenuation of the correlations between ordinal variables [32]. Before conducting the actual analysis, we checked the factorability of the items. Therefore, we examined whether the items were sufficiently correlated to produce representative factors by evaluating each item’s interitem correlation (>0.30, for at least three items), significance of Bartlett test of sphericity, and the Kaiser-Meyer-Olkin test (>0.80) [29,30].

We first conducted a principal component analysis to establish the number of factors to be extracted [27]. Then, we conducted a parallel analysis [33] with the eigenvalues as the leading criterion to determine the number of factors to extract, as this method is found to be the most robust and sophisticated [34]. As multiple criteria should be used to determine the number of factors to be extracted [29,35,36], we checked the results from the parallel analysis with other frequently used criteria: the Kaiser criterion (eigenvalue >1 [37]), the scree test (point on the scree plot where the eigenvalues seem to level off [38]), and the cumulative percentage of variance extracted (>60% explained variance [29]). However, this analysis did not yield different solutions.

For our common factor analysis, we applied the principal axis factoring method and Oblimin oblique rotation with Kaiser normalization. Oblique rotation is best suited in social sciences, as many constructs in this field have an empirically and theoretically based expectation to be correlated with each other [29,35,39]. Moreover, in the (unlikely) case that the factors are actually uncorrelated, orthogonal and oblique factors will produce similar results [40].

We cycled through an iterative analysis process [27,29,30]: (1) conduct a principal component analysis to determine the number of factors to retain; (2) conduct principal axis factoring with Oblimin rotation; (3) examine the rotated solution and assess the factor loadings for low loadings and cross-loadings; (4) evaluate potential items for deletion and delete the most problematic items. If an item was deleted, then we returned to step 1. This process was repeated until a satisfactory solution with a simple structure (ie, highest item loading >0.40, cross-loadings <0.30, and conceptual convergence between items in one factor) was achieved.

We then reverse scored the negatively worded items and obtained a total score, which was computed as the unweighted mean of all items, and obtained three subscale scores constituting the unweighted mean of the items of each of the three respective factors. We calculated both Pearson correlations and attenuation-corrected correlations between the subscales [41]. Finally, we calculated Cronbach *α* coefficients to probe the internal consistency.

To assess the construct validity of the scale, we examined convergent validity by calculating correlations between the scales (both the total scale and the subscales) and the measures we included for probing convergent validity: perceived added value of various EMH tools, feeling of competency regarding EMH, perceived proficiency regarding EMH skills and tools, and actual use of these tools. We calculated Spearman rank-order correlations because these items were measured on ordinal scales. It was expected that the total score would positively correlate with these additional measures and that such correlations would also be found for the subscale scores. All analyses were conducted using the Stata statistical software package, version 14.2 (StataCorp) [42].

### Validation Study 2

A second validation with a different sample was conducted to test whether the factor structure found in the first study could be confirmed and to find further support for the scale's psychometric properties.

#### Web-Based Survey

The second study used a web-based survey similar to the first study, comprising the resulting eMHAR Scale items from study 1 and the same convergent measures: perceived added value of EMH tools, feeling of competency regarding EMH, perceived proficiency in EMH skills and with specific tools, and frequency of use. We again expected that the eMHAR Scale and the subscales would correlate substantially with these concepts.

Furthermore, the survey included questions regarding barriers, drivers, and needs experienced by professionals while using EMH and questions on their experiences with the interaction with their clients through EMH. Owing to the outbreak of the COVID-19 pandemic, we also included some questions that probed differences in the use and perceived usefulness of EMH between the period before the COVID-19 pandemic and at the time of the survey (June-September 2020) during the first wave. The survey concluded with basic demographic questions, such as years of professional experience and whether they had received training in EMH. The required time to complete the survey was established at approximately 20 minutes.

#### Sample Size and Recruitment

To establish our required sample size, we followed a simulation study that compared the validity of confirmatory factor analysis solutions for various sample sizes [43]. For our 3-factor solution, the minimum required sample size to achieve an excellent agreement (ie, congruence values >0.98) between the sample and population solutions was determined to be 200. As in the first study, our recruitment was aimed at obtaining a sample representative of the population of mental health care professionals in the Netherlands. Four mental health care institutions disseminated an announcement with information and the survey link either through email or intranet. Furthermore, an announcement was placed in the newsletter of a national professional association of psychologists, that is, Dutch Mental Health care. The message contained information about the purpose of the study and the link to the web-based survey. Participants could sign up for a raffle in which 12 gift vouchers were allotted as a reward for their participation.

#### Data Collection and Sample

Data were collected between June 2020 and September 2020. It is important to note that this second phase of data collection occurred after the COVID-19 outbreak. Practitioners had been forced to switch to web-based treatments because face-to-face treatment was not, or only sparsely, possible during the first peak of the pandemic. This led to a sharp increase in the use of EMH, where 80%-90% of the practitioners never or only rarely made use of web-based tools in their routine [8-10]; in May 2020, 80%-90% reported using it almost daily [44]. In total, 363 participants (268/363, 73.8% female) completed the survey, with ages ranging from 18 to 70 years (mean 39.1, SD 11.5). Again, the most frequent professions were clinical or counseling psychologists, psychiatric nurses, and social workers. In total, 92.5% (336/363) of the respondents indicated that they did not participate in the previous study, and inclusion of the overlapping 7.4% (27/363) did not affect the results. Table 1 presents further details on the demographic data of the second sample.

#### Scale Analysis and Evaluation

After the data screening process as described for study 1, a confirmatory factor analysis was conducted on the data from the second sample with structural equation modeling using robust maximum likelihood estimation and the standardization of latent factors. The root mean square error of approximation (RMSEA) and the comparative fit index (CFI) were used to assess model fit; RMSEA<0.07 and CFI>0.92 indicated an acceptable model fit [29].

Then, we followed the same procedure as in study 1 to obtain the total score and subscale scores (ie, reverse scoring, computing the unweighted mean scores for the total scale and respective subscales), the correlations between the subscales, and Cronbach *α* coefficients. To assess the construct validity, we again calculated Spearman rank-order correlations between the scales and the associated measures to examine the convergent validity. Another important component of the scale’s validity pertains to its ability to measure changes in adoption readiness. Due to the sharp increase in the use of EMH as a consequence of the COVID-19 outbreak that occurred between the data collections [44], we expected that the gained experience in EMH would lead to higher feelings of competency in EMH; thus, scores on the EMH self-efficacy subscale and total scale should be higher in the second sample than in the first. No differences were expected in the other two subscales. We analyzed this through two methods of analysis: multigroup confirmatory factor analysis and independent samples two-tailed *t* tests. Although multigroup confirmatory factor analysis is a precise method to compare responses across groups, it depends on the particular samples that are being compared, which complicates cross-study comparisons, especially when complete data sets are not accessible [45]. Therefore, we also compared the unweighted means of the subscales with independent sample *t* tests. For the multigroup confirmatory factor analysis, the first step involved testing for various levels of measurement invariance, that is, whether the scale measured the same construct in the two samples, which is a precondition for comparing the sample means. We tested this using the free baseline approach, which means testing successive models that are gradually being restricted (a more elaborate description of this strategy is presented in the study by Kline [45]). A CFI decrease ≤0.01 was used as the criterion to indicate no significant decrement of model fit, that is, supporting measurement invariance across the samples [46]. After establishing measurement invariance, we compared the estimated mean differences. Here, we took study 1 as the reference group, setting the intercepts at 0, and the means of study 2 were free to estimate. The estimated mean differences are then assessed with the value of the critical ratio, which is calculated as the parameter estimate divided by its SE. This provides a *z* statistic that tests whether the estimate is statistically different from zero. Second, we also compared the unweighted mean scores on the total and subscales of the first and second sample using independent samples *t* tests (two-tailed, *α*=.05).

The (multigroup) confirmatory factor analysis was conducted with R, version 4.0 (R Foundation for Statistical Computing) [47], via the software package RStudio, version 1.3 (RStudio) using the lavaan library [48], which is a package for structural equation modeling. All other analyses were conducted using the Stata statistical software package, version 14.2 (StataCorp) [42].

## Results

### Validation Study 1

#### Exploratory Factor Analysis

Screening of the data showed that there were no missing values or violations of multivariate assumptions. We identified 11 cases out of the total sample of 432 that exceeded the critical value for the Mahalanobis distance. Upon inspection of these cases, none of them showed suspicious values (eg, only reporting very low or high values), suggesting that they misunderstood the items or had a particular response tendency. In addition, performing the factor analysis with and without these cases did not yield substantial differences in the found structure and factor loadings. Therefore, we decided to keep all the cases in our final analyses.

All 29 items had at least three interitem correlations >0.30. The Kaiser-Meyer-Olkin test of sampling adequacy had a value of 0.92 for the total item pool, and on the item level, all values exceeded 0.84. Bartlett test of sphericity (*χ*^2^_406_=7063.00) was significant (*P<*.001). These results indicated that the items were appropriate for factor analysis [29].

Initially, a 4-factor solution emerged from the iterative analysis procedure described above. During the process, we deleted seven items (in seven consecutive iterations) because the cross-loadings exceeded the recommended threshold of 0.30 [29]. From the remaining 21 items, four more items with relatively low loadings on all components were removed. One of the four factors then comprised only two items, both of which showed skewness values above the recommended value of 1.00 and were also difficult to interpret theoretically. For these reasons, these two items were also removed. The final 15-item solution with three factors explained 60.59% of the total variance. Table 2 shows the loadings of the items on the respective factors.

The first factor (items 1-7) concerns the perceived benefits and applicability of EMH; the extent to which the professional thinks EMH can have added value for clinical practice and fits the mental health care profession. The second factor (items 8-12) covers proactive innovation toward EMH; whether the professional encourages other colleagues to work with EMH and is involved in EMH development. The third factor (items 13-15) concerns perceived self-efficacy regarding EMH; the extent to which the professional feels competent and possesses the skills necessary to work with EMH.

**Table 2:** The 15 items in the final 3-factor solution and their factor loadings (>0.40)^a^.

Item	Factor 1: perceived benefits of EMH^b^	Factor 2: EMH proactive innovation	Factor 3: EMH self-efficacy
1. eHealth fits well to my work as a health care professional	0.67	—^c^	—
2. Contact between health care professional and client always has to be face-to-face (R^d^)	0.63	—	—
3. I expect that eHealth provides benefits to the care that I deliver	0.70	—	—
4. eHealth does not improve the care that I deliver (R)	0.71	—	—
5. eHealth does not fit the profession of a mental health care professional (R)	0.78	—	—
6. eHealth does not have any added value for my work as a mental health care professional (R)	0.69	—	—
7. eHealth is an indispensable part of the mental health care profession	0.54	—	—
8. I am involved in setting up initiatives for the development of new eHealth tools and applications	—	0.91	—
9. Compared with my colleagues, I use eHealth a lot	—	0.71	—
10. Compared with colleagues, I take a lot of initiative regarding eHealth	—	0.80	—
11. I have ideas about new eHealth tools and technologies that could be developed (eg, virtual reality, gaming, biofeedback)	—	0.59	—
12. In my work I try to stimulate colleagues to use eHealth	—	0.78	—
13. I have the skills that are necessary to apply eHealth in my work	—	—	0.71
14. Using eHealth tools comes easy to me	—	—	0.67
15. I have to learn new skills to start using eHealth (R)	—	—	0.63

^a^Extraction method; principal axis factoring; rotation method; Oblimin with Kaiser normalization.

^b^EMH: eMental health.

^c^Factor loading <0.3.

^d^R: reverse scored.

#### Psychometric Evaluation

Table 3 presents the descriptive statistics for the total scale and subscales. Multimedia Appendix 3 presents a histogram of the total scores.

**Table 3:** Descriptive statistics of the scores on the total eMHAR Scale and subscales in both studies.

Subscale		Mean (SD; range)	Median	Skewness	Kurtosis
**Study 1 (N=432)**					
	Total eMHAR^a^ Scale	3.22 (0.61; 1.60-5.00)	3.20	0.10	0.07
	Perceived benefits of EMH^b^	3.62 (0.64; 1.43-5.00)	3.71	−0.42	0.41
	EMH proactive innovation	2.65 (0.91; 1.00-5.00)	2.60	0.39	−0.32
	EMH self-efficacy	3.27 (0.77; 1.00-5.00)	3.33	−0.20	0.11
**Study 2 (N=363)**					
	Total eMHAR Scale	3.30 (0.60; 1.07-5.00)	3.33	−0.19	0.19
	Perceived benefits of EMH	3.65 (0.66; 1.00-5.00)	3.71	−0.55	0.49
	EMH proactive innovation	2.76 (0.88; 1.00-5.00)	2.80	0.08	−0.52
	EMH self-efficacy	3.42 (0.73; 1.00-5.00)	3.33	−0.51	0.70

^a^eMHAR: eMental Health Adoption Readiness.

^b^EMH: eMental health.

Internal consistency, calculated by Cronbach *α*, yielded satisfactory results for the total scale (.88) and the respective subscales (.73-.87, Table 4). The subscales were significantly correlated with each other (Table 4), suggesting that they share an underlying construct. Table 4 shows the Cronbach *α* values of the subscales, Pearson correlations, and corrected correlations between subscales.

**Table 4:** Internal consistencies and intercorrelations of the subscales.

Subscale			Perceived benefits of EMH^a^	EMH proactive innovation	EMH self-efficacy
**Study 1**					
	**Perceived benefits of EMH**				
		Coefficient	.83^b^	0.57^c^	0.45^c^
		*P* value	—^d^	<.001	<.001
	**EMH proactive innovation**				
		Coefficient	0.48^e^	.87^b^	0.58^c^
		*P* value	<.001	—	<.001
	**EMH self-efficacy**				
		Coefficient	0.35^e^	0.47^e^	.73^b^
		*P* value	<.001	<.001	—
**Study 2**					
	**Perceived benefits of EMH**				
		Coefficient	.86^b^	0.56^c^	0.37^c^
		*P* value	—	<.001	<.001
	**EMH proactive innovation**				
		Coefficient	0.48^e^	.87^b^	0.56^c^
		*P* value	<.001	—	<.001
	**EMH self-efficacy**				
		Coefficient	0.29^e^	0.45^e^	.74^b^
		*P* value	<.001	<.001	—

^a^EMH: eMental health.

^b^Reliability coefficient: Cronbach *α* (on diagonal).

^c^Correlation coefficients corrected for attenuation (above diagonal).

^d^Not applicable.

^e^Pearson correlations (below diagonal).

#### Convergent and Predictive Validity

To establish convergent validity, we analyzed correlations between the eMHAR Scale scores and constructs that have been associated with the adoption of EMH. As expected, the total mean score was positively correlated with all the relevant measures (Table 5). More specifically, a higher score on the eMHAR Scale was associated with higher perceived added value of EMH, higher feelings of competency in EMH, and higher perceived proficiency regarding various EMH skills and specific EMH tools. The scale also showed a predictive validity for the more frequent use of EMH tools.

We also explored the correlations between these measures and the three eMHAR subscales (Table 5). The strength of the relationship of the subscales corresponded to their respective intuitively related constructs: compared with the other subscales, the perceived benefits of the EMH subscale correlated highest with the perceived added value. In addition, the EMH self-efficacy subscale correlated highest with feeling of competency, perceived proficiency in various EMH skills, and specific EMH tools. EMH proactive innovation correlated the strongest with frequency of use. These results support the convergent validity of the eMHAR Scale. Importantly, the correlations with the score on the total scale were always higher than those with the scores on the individual subscales.

**Table 5:** Spearman rho correlations between scores on the total eMental Health Adoption Readiness Scale and the subscales, and related constructs in studies 1 and 2.

Subscale				Perceived added value		Feelings of competency		Perceived proficiency general		Perceived proficiency tools		Frequency of use
**Study 1**												
	**Total scale**											
		Correlation coefficient	0.54		0.63		0.69		0.47		0.50	
		*P* value	<.001		<.001		<.001		<.001		<.001	
	**Perceived benefits of EMH^a^**											
		Correlation coefficient	*0.48^b^*		0.38		0.48		0.26		0.38	
		*P* value	<.001		<.001		<.001		<.001		<.001	
	**EMH proactive innovation**											
		Correlation coefficient	0.44		0.56		0.57		0.45		*0.47*	
		*P* value	<.001		<.001		<.001		<.001		<.001	
	**EMH self-efficacy**											
		Correlation coefficient	0.31		*0.58*		*0.64*		*0.46*		0.30	
		*P* value	<.001		<.001		<.001		<.001		<.001	
**Study 2**												
	**Total scale**											
		Correlation coefficient	0.43		0.57		0.66		0.38		0.39	
		*P* value	<.001		<.001		<.001		<.001		<.001	
	**Perceived benefits of EMH**											
		Correlation coefficient	*0.40*		0.34		0.46		0.21		0.27	
		*P* value	<.001		<.001		<.001		<.001		<.001	
	**EMH proactive innovation**											
		Correlation coefficient	0.37		0.51		0.55		0.35		*0.40*	
		*P* value	<.001		<.001		<.001		<.001		<.001	
	**EMH self-efficacy**											
		Correlation coefficient	0.16		*0.55*		*0.64*		*0.36*		0.22	
		*P* value	.002		<.001		<.001		<.001		<.001	

^a^EMH: eMental health.

^b^Italics indicate correlations of the highest scoring subscale.

### Validation Study 2

#### Confirmatory Factor Analysis

Following the same data screening process as in study 1, no missing values or violations of assumptions were found. In total, 11 cases out of the total sample of 363 exceeded the critical value for the Mahalanobis distance, but their responses again showed no suspicious values. Performing the factor analysis with and without these cases yielded almost identical results for the factor structure and loadings. On the basis of this result, all cases were retained for our final analyses.

The confirmatory factor analysis confirmed the 3-factor model with 15 items found in study 1, showing relatively good fit indices (*χ*^2^_87_=200.4, *P*<.001; CFI=0.96; RMSEA=0.062). The model provided satisfactory standardized factor loadings (ie, >0.40) for all items on their respective factors, as shown in Table 6.

**Table 6:** Standardized factor loadings (>0.40) of the 15 items in the 3-factor model.

Item	Factor 1: Perceived benefits of EMH^a^	Factor 2: EMH proactive innovation	Factor 3: EMH self-efficacy
1. eHealth fits well to my work as a health care professional	0.81	—^b^	—
2. Contact between health care professional and client always has to be face-to-face (R^c^)	0.68	—	—
3. I expect that eHealth provides benefits to the care that I deliver	0.71	—	—
4. eHealth does not improve the care that I deliver (R)	0.73	—	—
5. eHealth does not fit the profession of a mental health care professional (R)	0.73	—	—
6. eHealth does not have any added value for my work as a mental health care professional (R)	0.64	—	—
7. eHealth is an indispensable part of the mental health care profession	0.50	—	—
8. I am involved in setting up initiatives for the development of new eHealth tools and applications	—	0.71	—
9. Compared to my colleagues, I use eHealth a lot	—	0.82	—
10. Compared to colleagues, I take a lot of initiative regarding eHealth	—	0.87	—
11. I have ideas about new eHealth tools and technologies that could be developed (eg, Virtual Reality, gaming, biofeedback)	—	0.60	—
12. In my work I try to stimulate colleagues to use eHealth	—	0.80	—
13. I have the skills that are necessary to apply eHealth in my work	—	—	0.77
14. Using eHealth tools comes easy to me	—	—	0.99
15. I have to learn new skills to start using eHealth (R)	—	—	0.41

^a^EMH: eMental health.

^b^Factor loading <0.3.

^c^R: reverse scored.

#### Psychometric Evaluation

Table 3 presents the descriptive statistics for the total scale and subscales. Multimedia Appendix 4 presents a histogram of the total scores.

Regarding the reliability of the questionnaire, measured by Cronbach *α*, the values found for the total scale (.88) and the respective subscales (.74-.87, Table 4) were almost identical to those in study 1, again providing evidence for satisfactory internal consistency. In addition, similar values were found for the intercorrelations between the subscales (Table 4), which were all significantly correlated with each other, further confirming that they share an underlying construct.

#### Convergent and Predictive Validity

Convergent validity was again evaluated by analyzing the correlations between the eMHAR Scale scores and associated constructs. As expected, the total score correlated positively with all the relevant measures (Table 5). More specifically, a higher score on the eMHAR Scale was associated with a higher perceived added value of EMH, higher feeling of competency for EMH, and higher perceived proficiency in EMH skills and for EMH tools. The scale also showed predictive validity for the more frequent use of EMH tools.

Next, we analyzed the correlations between these measures and the three eMHAR subscales (Table 5), which yielded the expected results, similar to study 1: compared with the other subscales, the perceived benefits of the EMH subscale correlated highest with the perceived added value, the self-efficacy subscale correlated highest with the feeling of competency, proficiency in general EMH skills, and for specific tools. EMH proactive innovation correlated the strongest with the frequency of use. These results confirmed the validity of the subscales.

Finally, we assessed the scale’s sensitivity to detect changes in adoption readiness by comparing the total and subscale scores between the two samples, as changes were expected because of the increased EMH use following the COVID-19 pandemic. After establishing measurement invariance (see Multimedia Appendix 5 for the goodness-of-fit test statistics), the multigroup confirmatory factor analysis showed a significant estimated mean increase of 0.25 units between study 1 and study 2 for EMH self-efficacy (*z*=3.342; *P*=.001) but not for the other factors (perceived benefits of EMH: *z*=0.543; *P*=.59; and EMH proactive innovation: *z=*1.862; *P*=.06), all in line with our expectations. The same results were found while comparing the unweighted mean scores for the total scale and subscales; we found significant differences for the EMH self-efficacy subscale (*t_793_*=−2.79; *P=*.005) and a trend for the total scale (*t_793_*=−1.87; *P*=.06) but not for the perceived benefits of the EMH subscale (*t_793_*=−0.60; *P*=.55) or the EMH proactive innovation subscale (*t_793_*=−1.76; *P*=.08).

## Discussion

### Principal Findings

EMH has been shown to offer many promising possibilities for mental health care delivery [4-7], as also recognized by many clients [49]. Despite this, before the COVID-19 pandemic, the adoption of EMH technologies by mental health care professionals remained low [8-10]. To facilitate research on the adoption of EMH, we set out to develop a valid instrument that can reliably assess a professional’s readiness to adopt EMH, is independent of organizational setting and specific tools, and can be easily applied to larger groups. To date, such an instrument did not exist. The eMHAR Scale was based on a thorough consultation of both literature and professionals in the field. Initial testing with a large sample of the target population provided a meaningful 3-factor structure with good internal consistency and convergent validity. The structure and satisfactory psychometric properties were confirmed in a second sample, thereby supporting the scale’s ability to assess adoption readiness for the EMH of mental health care professionals.

Investigating the underlying structure of the eMHAR Scale indicated the existence of three factors: perceived benefits and applicability of EMH, EMH proactive innovation, and EMH self-efficacy. Identifying these underlying components allows us to look at the determinants that are important for the adoption of EMH in a new way, providing information on how they are connected to each other, which aspects are general to the adoption of technologies, and which aspects are more specific to the field of mental health care.

For the first factor, perceived benefits and applicability of EMH, some of the items are similar to items in factors of previous models on the adoption of novel technologies (eg, the factor Performance Expectancy in the unified theory of acceptance and use of technology model [50]), indicating that the perception that a new technology adds value is a fundamental factor in its adoption. These items on perceived benefits in the eMHAR Scale were highly related to the items regarding the extent to which practitioners feel EMH is appropriate for mental health care delivery. This is in line with other research on the adoption of innovations in health care that reported that the innovation’s compatibility with the practitioner’s profession is an important determinant of its adoption [51]. The emergence of this factor with items of both topics suggests that the applicability of the technologies to care delivery constitutes an essential part of perceiving benefits for (mental) health care professionals, which seems to differentiate the adoption of health care technologies from consumer technologies.

The second factor, EMH proactive innovation, includes some items that resemble the construct of personal innovativeness, defined as the willingness of individuals to try out a new technology [52]. However, in the eMHAR Scale, these items were related to the items of proactive behavior such as being involved in EMH initiatives and stimulating the use of EMH among colleagues. The social aspect of these items bears some resemblance with the social influence factor of the unified theory of acceptance and use of technology model [50], but those items are mainly focused on using a technology to adhere to a subjective norm. This could indicate that in mental health care, the subjective norm is less important for the adoption of technology, whereas adoption is more influenced by a person's individual degree of proactivity regarding technologies.

The third factor concerns EMH’s self-efficacy, that is, the feeling that you possess the necessary skills and knowledge to use EMH. Self-efficacy has been identified as a common determinant in research on the adoption of innovations in health care [51] as well as in other areas (eg, learning technologies [53]). It has also been found to be an important predictor of technology use in general [54] and for specific technologies such as computers [55]. Furthermore, research indicates that self-efficacy influences both behavior and evaluation; when someone's perceived ability to use a new technology increases, this has a positive effect on both actual use and how this use is evaluated [56]. Thus, it can be expected that higher levels of EMH self-efficacy are related to a more frequent use of EMH tools and to experiencing more benefits, in line with our findings that the EMH self-efficacy subscale showed substantial positive correlations with the use of EMH tools and with the perceived benefits and applicability of the EMH subscale. It should be noted that although the three identified factors showed these intercorrelations, the total score showed the strongest correlations with the convergent and predictive measures (ie, use of EMH tools), which suggests that the total score for adoption readiness might be the most informative measure.

### Implications

The main goal of the eMHAR Scale is to measure professionals' readiness to adopt EMH and its underlying factors (ie, perceived benefits of EMH, EMH proactive innovation, and EMH self-efficacy). As these individual characteristics of professionals play a crucial role in the adoption process [12], acquiring a deeper understanding of adoption readiness is essential to truly grasp the intricacies of this process. There are several ways in which the eMHAR Scale could be used to achieve this goal. First, the scale could be used for descriptive purposes, providing knowledge on the current level of adoption readiness and its specific factors. These measurements could be used at both the individual and group levels, for example, within a particular organization or country. In addition, such assessments could concern a single measurement at a particular moment or they could be used in longitudinal studies to assess changes over time by repeating measurements at predetermined intervals.

Second, the scale could facilitate the development and assessment of interventions aimed at changing EMH adoption, such as a specific training program. The scale—and its subscales—could indicate to organizations which specific factors are most opportune to target and in this way inform strategies to increase use of EMH in daily practice, as opposed to the one-size-fits-all approach that is currently standard practice [57]. For example, finding a relatively low score on the EMH self-efficacy subscale suggests that it might be worthwhile to focus on increasing skills and knowledge by organizing training on EMH skills, whereas a low score on perceived benefits of EMH suggests that actions could be taken to increase the awareness of their merits and advantages, or that perhaps the available EMH tools require improvement or refinement. In addition to informing the development of interventions, it could also be used to examine the effects of these interventions by comparing pre- and postintervention measurements.

Finally, the scale could contribute to more explanatory studies that aim to obtain a more in-depth understanding of EMH adoption by investigating how adoption readiness relates to other constructs or how it relates to different clinical contexts and therapeutic interventions. For example, studies could investigate relationships between scores on the eMHAR Scale and its subscales with factors such as gender and age, client population, (digital) skills, education, type of mental health care organization and profession, and specific EMH tools. In addition, scholars could examine the relationship between adoption readiness and actual use and the extent to which demographic or external factors influence this relationship. It would also be interesting to investigate how adoption readiness scores can be related to experienced barriers, drivers, and needs that have been reported in previous studies. Knowing how these experiences are influenced by the determinants of adoption readiness and actual use might provide a better understanding of why and under which circumstances they arise. In addition, the inclusion of this instrument across multiple studies will allow for comparison across different groups and settings, which facilitates a more cumulative science of the adoption of EMH tools.

While applying the eMHAR Scale, it is important to note that the scale is not intended to be used as a normative scale; a higher score is not per definition a better score. It is meant to provide a view of professionals’ current status regarding their readiness to adopt EMH. Furthermore, the level of adoption readiness does not correspond one-to-one with actual use in practice (and hence, is not a direct measure of adoption according to our terminology). Although we found a substantial positive correlation, meaning that practitioners with higher eMHAR scores were more likely to use EMH than those with lower scores, actual use in practice is also influenced by several external factors, such as technical infrastructure and organizational support [11-13]. Hence, this should be considered while aiming to increase the actual use of EMH.

### Strengths and Limitations

Although there were no established instruments that could be used to test the validity of the scale, the convergent and predicted measures showed expected relationships with the eMHAR Scale, supporting its external validity. In addition, finding the expected increase in scores on the EMH self-efficacy subscale between the two samples (pre-COVID-19 and a few months after the first lockdown) provided evidence for the sensitivity of the scale. A drawback associated with the unfolding of the COVID-19 pandemic and its associated physical distancing measures (eg, lockdown) that occurred between the two studies is the fundamental change in daily clinical practice that happened as a consequence [44]; therefore, a valid measure of test-retest reliability could not be established. As it is expected that the position of EMH in mental health care practice will continue to evolve over the coming years, a strict test-retest assessment will probably remain difficult to conduct in future work. On the other hand, the fact that the factor structure remained unchanged, even during this radical change in care practice, speaks to the robustness of the scale. We are also aware that the correlational and descriptive nature of this study design limits us in drawing causal inferences. Performing a study that applies the scale before and after an intervention for EMH adoption (eg, a specific training or providing guidelines for web-based treatment) might provide a more solid test for its sensitivity.

An important additional asset of this instrument is that it can be applied relatively quickly and unobtrusively, both on the internet and offline, thereby facilitating the systematic study of EMH adoption readiness. This is especially important for application in mental health care practice, where practitioners generally work under high time pressure, and experience the challenge of continuously delivering high-quality care to a growing population of people in need of care, while dealing with changing job demands in mental health care. Another asset is the scale’s independence in organizational setting and specific technologies. Therefore, it is not restricted to the mental health care systems of particular countries or practitioners working in institutions, and its applicability will not be compromised by future technological developments.

A strength of the current validation studies in support of the scale’s validity is that the building and testing of the model were conducted using two substantial samples (exceeding the recommended 10 participants per item [29,30]), and particularly that the results were robust despite the great change in the daily practice of mental health care that occurred during the time frame of the research. Another strength of the validation studies is that both samples consisted of mental health care professionals with a high variation in use and experience with EMH, professions within mental health care, and types of mental health care institutions. This benefits the generalizability of the sample toward the entire population of mental health care professionals in the Netherlands, and perhaps to similar countries, and thereby the generalizability of the results of the study across clinical approaches and contexts. Currently, the scale has only been examined in the Dutch language. Translation to other languages would allow for testing the validity of the scale internationally. As translation might lead to subtle differences in participants' responses (eg, subtle differences in semantics of terms may lead to a different interpretation of items), we plan to investigate whether the same factor structure will emerge. These translated scales could then be used to study whether findings can be generalized across other countries, including those with significantly different mental health care systems and technological infrastructures.

### Conclusions

This paper presents the construction and validation of the eMHAR Scale, a measurement instrument to assess the EMH adoption readiness of mental health care professionals. Overall, the scale showed satisfactory characteristics and relationships with relevant concepts. These results suggest that the eMHAR Scale is robust, valid, and reliable. With this work, we aim to stimulate future research and use in practice, which we hope will lead to improved insights into the individual characteristics of professionals in adopting EMH and facilitate well-informed solutions for the adoption process from which professionals, clients, and mental health care as a whole can benefit.

## Appendix

Multimedia Appendix 1 The total item pool in Dutch and English translation (items included in the final 15-item solution are italicized; reversed scoring items are indicated with R).

Multimedia Appendix 2 Survey items for convergent measures (translated from Dutch).

Multimedia Appendix 3 Histogram of the total scores in study 1.

Multimedia Appendix 4 Histogram of the total scores in study 2.

Multimedia Appendix 5 Tests of measurement invariance for the multigroup measurement model of the eMHAR Scale across the two studies.

## Abbreviations

CFI: comparative fit index
EMH: eMental health
eMHAR: eMental Health Adoption Readiness
RMSEA: root mean square error of approximation
