# Supplementary material for: Assessing Professionals’ Adoption Readiness for eMental Health: Development and Validation of the eMental Health Adoption Readiness Scale
Source: J Med Internet Res. 2021 Sep 17;23(9):e28518. doi: 10.2196/28518 (PMC8486999; doi:10.2196/28518)
Supplement: Multimedia Appendix 1 [file jmir_v23i9e28518_app1.docx]

## Appendix 1

Total item pool in Dutch and English translation (items included in the final 15-item solution are italicized, reversed scoring items are indicated with R).

| Item no. | Dutch | English |
| --- | --- | --- |
| *1* | *eHealth sluit goed aan bij mijn werk als zorgverlener.* | *eHealth fits well to my work as a healthcare professional.* |
| 2R | *Contact tussen zorgverlener en cliënt hoort altijd face to face te zijn.* | *Contact between healthcare professional and client always has to be face-to-face.* |
| *3* | *Ik verwacht dat eHealth voordelen biedt voor de zorg die ik verleen.* | *I expect that eHealth provides benefits to the care that I deliver* |
| *4* | *Ik houd me bezig met het opzetten van initiatieven voor de ontwikkeling van nieuwe eHealth toepassingen.* | *I am involved in setting up initiatives for the development of new eHealth tools and applications.* |
| *5R* | *eHealth biedt geen verbetering aan de zorg die ik verleen* | *eHealth does not improve the care that I deliver* |
| *6* | *Vergeleken met collega's maak ik veel gebruik van eHealth* | *Compared to my colleagues, I use eHealth a lot.* |
| 7 | Ik ben terughoudend in het inzetten van eHealth | I am reluctant to use eHealth |
| 8R | Ik gebruik eHealth toepassingen die gemakkelijk zijn in het gebruik. | I use eHealth applications that are easy to use |
| *9R* | *eHealth past niet bij het beroep van zorgverlener* | *eHealth does not fit the profession of a mental healthcare professional* |
| *10* | *Ten opzichte van collega’s neem ik veel initiatief op het gebied van eHealth* | *Compared to colleagues, I take a lot of initiative regarding eHealth* |
| *11* | *Ik bezit vaardigheden die nodig zijn om eHealth toe te passen in mijn werk.* | *I have the skills that are necessary to apply eHealth in my work.* |
| 12 | Ik weet niet hoe ik eHealth in kan passen in de zorg die ik verleen | I do not know how I can incorporate eHealth in the care that I deliver |
| 13 | Ik maak het liefst zo min mogelijk gebruik van de computer en andere nieuwe technologieën. | I prefer to use computers and other novel technologies as little as possible. |
| *14* | *Het gebruiken van eHealth toepassingen gaat me gemakkelijk af.* | *Using eHealth tools comes easy to me* |
| *15R* | *Om eHealth te gaan gebruiken moet ik nieuwe vaardigheden leren* | *I have to learn new skills to start using eHealth.* |
| 16 | Het gebruiken van eHealth in mijn werk is voor mij een gewoonte. | It is a habit for me to apply eHealth in my daily practice. |
| *17R* | *eHealth heeft geen toegevoegde waarde voor mijn werk als zorgverlener* | *eHealth does not have any added value for my work as a mental healthcare professional* |
| 18 | eHealth zou op een grotere schaal ingezet moeten worden. | eHealth should be implemented on a large scale |
| 19 | Het gebruiken van eHealth zit niet in mijn systeem | Using eHealth is not part of my daily routine |
| 20 | Ik heb ervaren dat eHealth de kwaliteit van de zorg die ik verleen ten goede komt. | I experienced that eHealth is beneficial to the care that I deliver. |
| *21* | *eHealth is een onmisbaar onderdeel van het werk van een zorgverlener* | *eHealth is an indispensable part of the mental healthcare profession* |
| *22* | *Ik heb ideeën over wat er nog meer ontwikkeld zou kunnen worden aan eHealth toepassingen (bijv. toepassing virtual reality, gaming, biofeedback)* | *I have ideas about new eHealth tools and technologies that could be developed (e.g. Virtual Reality, gaming, biofeedback)* |
| 23 | In de dagelijkse praktijk is het geen automatisme om eHealth in te zetten in de zorg die ik verleen | In daily practice I do not automatically incorporate eHealth in the care that I deliver. |
| 24R | Ik gebruik eHealth toepassingen die eenvoudig voor handen zijn | I use eHealth applications that are easily available to me. |
| 25 | Ik heb geen ervaring met het inzetten van eHealth. | I have no experience with using eHealth. |
| *26* | *In mijn werk probeer ik collega’s aan te sporen eHealth te gebruiken.* | *In my work I try to stimulate colleagues to use eHealth.* |
| 27 | eHealth heeft voor mij een lage prioriteit | eHealth has a low priority for me |
| 28 | Ik maak dagelijks gebruik van eHealth in mijn werk | I make use of eHealth on a daily basis |
| 29 | Ik ben geïnteresseerd in nieuwe ontwikkelingen en toepassingen om eHealth te gebruiken in mijn werk als zorgverlener | I am interested in new developments and application for using eHealth in my work as a mental healthcare professional |
