# Supplementary material for: Assessing Professionals’ Adoption Readiness for eMental Health: Development and Validation of the eMental Health Adoption Readiness Scale
Source: J Med Internet Res. 2021 Sep 17;23(9):e28518. doi: 10.2196/28518 (PMC8486999; doi:10.2196/28518)
Supplement: Multimedia Appendix 2 [file jmir_v23i9e28518_app2.docx]

## Appendix 2

### Survey items for the convergent measures (translated from Dutch).

#### Perceived added value

To which extent do you feel that applying the following eHealth tools could be of added value in the treatment of your client?

|  | Not at all valuable | Very little valuable | Somewhat  valuable | Valuable | Very valuable |
| --- | --- | --- | --- | --- | --- |
| E-mail |  |  |  |  |  |
| Whatsapp/text messaging |  |  |  |  |  |
| Client portal |  |  |  |  |  |
| Online modules in an eHealth platform |  |  |  |  |  |
| Social media (e.g., Facebook) |  |  |  |  |  |
| Home automation/Domotics (assistive technologies at home) |  |  |  |  |  |
| Wearables and biofeedback |  |  |  |  |  |
| Virtual/Augmented Reality |  |  |  |  |  |
| 360 degrees camera |  |  |  |  |  |
| Selfmonitoring apps (e.g., sleep diary) |  |  |  |  |  |
| Videoconferencing |  |  |  |  |  |
| Online screening |  |  |  |  |  |
| Informational website |  |  |  |  |  |

#### Frequency of Use

How often, on average, do you make use of the following eHealth tools in the treatment of your client?

|  | (Almost) never | Approx. 1-2 times per half year | Approx. 1-2 times per month | Approx. 1-2 times per week | (Almost) every day |
| --- | --- | --- | --- | --- | --- |
| E-mail |  |  |  |  |  |
| Whatsapp/text messaging |  |  |  |  |  |
| Client portal |  |  |  |  |  |
| Online modules in an eHealth platform |  |  |  |  |  |
| Social media (e.g., Facebook) |  |  |  |  |  |
| Home automation/Domotics (assistive technologies at home) |  |  |  |  |  |
| Wearables and biofeedback |  |  |  |  |  |
| Virtual/Augmented Reality |  |  |  |  |  |
| 360 degrees camera |  |  |  |  |  |
| Selfmonitoring apps (e.g., sleep diary) |  |  |  |  |  |
| Videoconferencing |  |  |  |  |  |
| Online screening |  |  |  |  |  |
| Informational website |  |  |  |  |  |

#### Feelings of competency

Could you indicate how competent you feel in general regarding the application of eHealth tools? Please choose the statement which best describes your feeling of competency:

- I have insufficient knowledge of which eHealth tools are available.
- I have a notion of the available eHealth tools, but I do not know how to integrate them in daily practice.
- I know how to use some of the eHealth tools and how to deploy their basic functionalities in daily practice.
- I am able to figure out on my own how eHealth tools work and therefore I can work well with various different eHealth tools.
- I am very familiar with a range of different eHealth tools and able to transfer my knowledge and expertise in this area to others.

#### Perceived proficiency of EMH in general

The following statements concern your feelings of proficiency regarding eHealth. Please indicate for the following statements to which extent you feel they apply to you:

|  | Strongly disagree | Disagree | Neutral | Agree | Strongly agree |
| --- | --- | --- | --- | --- | --- |
| I have sufficient knowledge about the available options in the area of eHealth to be able to use eHealth tools. |  |  |  |  |  |
| I have sufficient ICT- and computer skills to use eHealth tools. |  |  |  |  |  |
| I have sufficient knowledge and skills to proactively offer eHealth to my clients. |  |  |  |  |  |
| I can communicate in a satisfactory way with my clients when using eHealth. |  |  |  |  |  |
| I am aware of the security issues and tradeoffs (e.g., privacy and personal data protection) of using eHealth in the contacts with my clients. |  |  |  |  |  |
| I can handle well that the application of eHealth puts the client more in control of the content, frequency, mode, and location of the treatment contact. |  |  |  |  |  |
| I can successfully establish an empathic interaction with my client online. |  |  |  |  |  |
| I have sufficient knowledge about the available options in the area of eHealth to be able to use eHealth tools. |  |  |  |  |  |
| I have sufficient ICT- and computer skills to use eHealth tools. |  |  |  |  |  |
| I have sufficient knowledge and skills to proactively offer eHealth to my clients. |  |  |  |  |  |
| I can communicate in a satisfactory way with my clients when using eHealth. |  |  |  |  |  |
| I am aware of the security aspects and issues (e.g., privacy and personal data protection) of using eHealth in the contacts with my clients. |  |  |  |  |  |

#### Perceived proficiency of EMH of eHealth tools

Please indicate for each of these eHealth tools how proficient you feel in using them in the treatment of your clients.

|  | Not at all proficient | Very little proficient | Somewhat proficient | Proficient | Very proficient |
| --- | --- | --- | --- | --- | --- |
| E-mail |  |  |  |  |  |
| Whatsapp/text messaging |  |  |  |  |  |
| Client portal |  |  |  |  |  |
| Online modules in an eHealth platform |  |  |  |  |  |
| Social media (e.g., Facebook) |  |  |  |  |  |
| Home automation/Domotics (assistive technologies at home) |  |  |  |  |  |
| Wearables and biofeedback |  |  |  |  |  |
| Virtual/Augmented Reality |  |  |  |  |  |
| 360 degrees camera |  |  |  |  |  |
| Selfmonitoring apps (e.g., sleep diary) |  |  |  |  |  |
| Videoconferencing |  |  |  |  |  |
| Online screening |  |  |  |  |  |
| Informational website |  |  |  |  |  |
