# Supplementary figures and images for: Assessing Professionals’ Adoption Readiness for eMental Health: Development and Validation of the eMental Health Adoption Readiness Scale
Source: J Med Internet Res. 2021 Sep 17;23(9):e28518. doi: 10.2196/28518 (PMC8486999; doi:10.2196/28518)

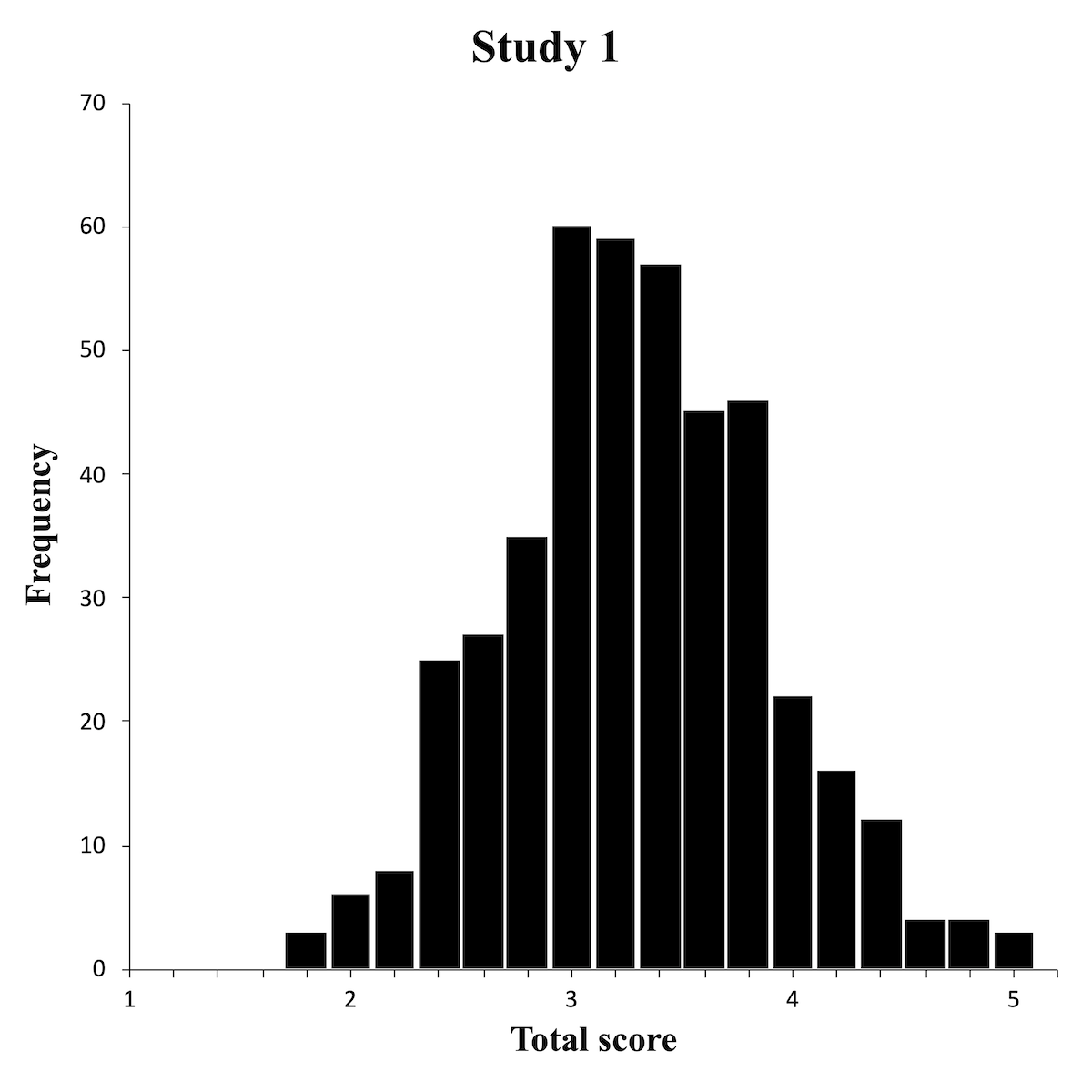

Supplement: Multimedia Appendix 3 [file jmir_v23i9e28518_app3.png]

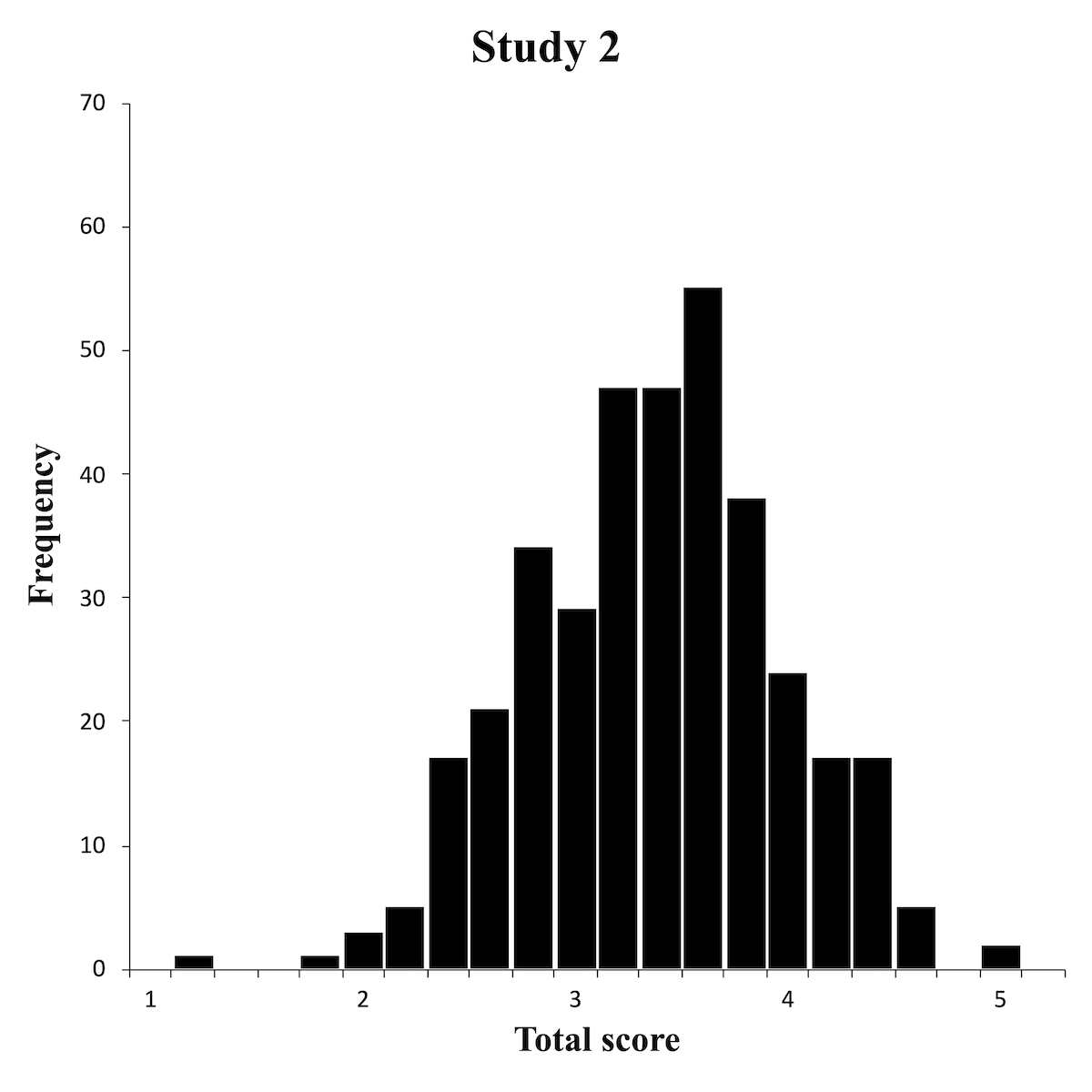

Supplement: Multimedia Appendix 4 [file jmir_v23i9e28518_app4.png]
