# Supplementary material for: Assessing Professionals’ Adoption Readiness for eMental Health: Development and Validation of the eMental Health Adoption Readiness Scale
Source: J Med Internet Res. 2021 Sep 17;23(9):e28518. doi: 10.2196/28518 (PMC8486999; doi:10.2196/28518)
Supplement: Multimedia Appendix 5 [file jmir_v23i9e28518_app5.docx]

## Appendix 5

Tests of measurement invariance for the multi-group measurement model of the eMHAR Scale across the two studies.

| Model | $\chi$^2^ | df | CFI | $\Delta$CFI (with previous model) | RMSEA | SRMR |
| --- | --- | --- | --- | --- | --- | --- |
|  |  |  |  |  |  |  |
| Model 1^a^ | 498.293 | 174 | 0.939 |  | 0.068 | 0.050 |
| Model 2^b^ | 515.606 | 186 | 0.938 | 0.001 | 0.067 | 0.055 |
| Model 3^c^ | 566.564 | 198 | 0.931 | 0.007 | 0.068 | 0.057 |
| Model 4^d^ | 629.691 | 213 | 0.922 | 0.009 | 0.070 | 0.058 |

^a^Configural invariance model (no constraints)

^b^Metric invariance model (equal loadings)

^c^Scalar invariance model (equal loadings and intercepts)

^d^Strict invariance model (equal loadings, intercepts, and residuals)
